# Process of Deinstitutionalization of Aging Individuals With Severe and Disabling Mental Disorders: A Review

**DOI:** 10.3389/fpsyt.2022.813338

**Published:** 2022-03-24

**Authors:** Samira Salime, Christophe Clesse, Alexis Jeffredo, Martine Batt

**Affiliations:** ^1^INTERPSY Laboratory, University of Lorraine, Nancy, France; ^2^Hope 54 Association, Nancy, France; ^3^Center for Psychiatry, Wolfson Institute of Preventive Medicine, Barts and The London School of Medicine Dentistry, Queen Mary University of London, London, United Kingdom; ^4^Psychiatric Hospital of Jury-les-Metz, Metz, France

**Keywords:** deinstitutionalization, severe mental disorder, aging, elderly, transinstitutionalization, psychiatry

## Abstract

**Background:**

For more than 60 years, psychiatric services has gradually gone from an asylum model to a community model. This change has led to the emergence of a deinstitutionalization movement. This movement seems to have left behind long-term hospitalized aging individuals with severe and disabling mental disorders. The objective of this article is to conduct a review on the challenges and issues associated with the process of deinstitutionalization among hospitalized aging individuals with severe and disabling mental disorders.

**Methods:**

Using PRISMA statement, the research methodology was carried out in English and French in 16 databases with a combination of 3 lists of keywords. The selection process was then followed by a thematic analysis which aimed at categorizing by theme and classifying the writings selected.

**Results:**

A total of 83 articles published between 1978 and 2019 were selected and organized into six categories: (a) a forgotten population in research and health policies, (b) an economic presentation of the deinstitutionalization process, (c) an improvement in quality of life and global functioning for deinstitutionalized patients (d) from stigmatization to the rejection of elderly psychiatric inpatients from deinstutionalization process, (e) a difficult community-based care offer and a difficult epistemological identification, (f) from the lack of community services to the phenomenon of transinstitutionalization. The current state of scientific research, institutional policies and clinical practices associated with the deinstitutionalization process of SVPTSIH are then commented.

**Conclusions:**

Recommendations are proposed to researchers and professionals concerned with the support of long-term hospitalized aging individuals with severe and disabling mental disorders.

## Background

For more than 60 years, the treatment of mental disorders in Western countries has been driven by profound changes. Under the influence of numerous political, ideological, economic, and scientific factors (the development of new neuroleptics or the dissemination of new therapeutic methods, etc.), the vision of psychiatric care for individuals suffering from severe and disabling mental disorders has been profoundly modified (1). Previously oriented toward an asylum-centered approach centralizing support within the hospital walls, psychiatric monitoring has gradually turned toward a community approach that potentiates the development of ambulatory structures anchored in the city (1–3).

To this end, many psychiatric structures have initiated a deinstitutionalization movement defined as “a complex process in which a reduction in psychiatric beds is associated with the implementation of community-based alternatives aimed at avoiding the internment of individuals suffering from psychological pathologies” (4). Within a structure dedicated to day care, outpatient mental health services or through supported housing (often supported by the work of a psychosocial rehabilitation team), many individuals have been able to benefit from support anchored in the social fabric, the benefits of which have been commented on in the scientific literature (3, 5–10).

Nowadays, it seems that aging people with severe and disabling mental health disorders who are hospitalized for long periods of time (APSDMHD-H) do not fully benefit from this mechanism (8, 11, 12). Yet, the numerical importance of this population (13, 14), the difficulty of institutions to provide an appropriate ambulatory care setting for these individuals (15–17), or the little emergence of new knowledges in this field (18) shows today the need and urgency to conduct extensive research work in this specific field.

In this context, the authors establish a complete inventory of all the scientific literature on the process of deinstitutionalization of individuals with severe and disabling mental disorders. The aim of this work is to present all the scientific productions in this field to isolate, present and comment the knowledge already acquired in the field of deinstitutionalization of the elderly patients in a situation of psychological or mental handicap. Similarly, this narrative review of the literature based on a systematic search will aim to identify unexplored areas of work while proposing a reflection on the reasons associated with these possible shortcomings. Finally, we propose recommendations aimed at outlining the axes necessary for the implementation of a future research strategy on the problems of this specific population.

## Methods

Relying on PRISMA statement (19, 20), this narrative review based on a systematic search (Prospero ID: CRD42020158689) provides a comprehensive scientific review of the deinstitutionalization of elderly individuals with mental disorders. To do so, between July and August 2019, the authors selected all the scientific articles available on the 16 following databases: “*Public Health Database, CAIRN, Sage, Cochrane, Embase, JSTOR, Psycinfo and Psycarticles, Pubmed (Medline), Biomed, Science Direct, Springer, Taylor* & *Francis, Wiley, and Web of Science, Open-edition.”* This research has been then updated in September 2021.

Authors isolated, regarding the scope of this review and inclusion criteria, all scientific articles that refers to elderly inpatients in deinstitutionalization process. We excluded all the book chapters, conference abstracts, editorials, and short comments. The article selection was not based on a specific publication period. To perform the selection of articles, the authors used a combination of three lists of key words in French and English. The first list of key word is: “*Désinstitutionalisation”/“desinstitutionalization”; “institutionnalisation”/“institutionalization”; “Réhabilitation psychosociale”/“Psychosocial rehabilitation”; “empowerment.”* The second list of keywords is: “*Personne âgée”/“elderly”; “vieillissement”/“aging”; “vieillesse”/“old people”; “geriatrics.”* Finally, the third list of key words is: “*psychic disorders,” “psychosis,” “psychic disability,” “psychiatry,” “mental disorder,” “mental disability,” “psychical disability,” “psychosis,” “mental disorder,” “chronic psychosis,” “schizophrenia.”*

The process of article selection was carried out in a double-blind manner by the first two authors of the article and, in the event of disagreement; a joint reflection was undertaken with the third author of the article. Depending on the specificities of the databases, the authors carried out their search based on title, abstract and keywords (*Pubmed, Science Direct, Web of Science, Embase, Cochrane*) and on the whole text (*Public Health Database, CAIRN, Biomed, JSTOR, SAGE, Psycinfo, Psycarticle, Springer, Taylor and Francis, Wiley*). Thus, among the selection proposed by all the databases (22,642) we retained 1,010 articles from their title read. We then added five references from gray literature (reports, book chapters, book) and removed duplicates. We read the remaining 988 abstract and excluded 894 papers. At last, we fully read 90 articles leading to a rejection of 7 out of scope articles.

**Figure 1:**
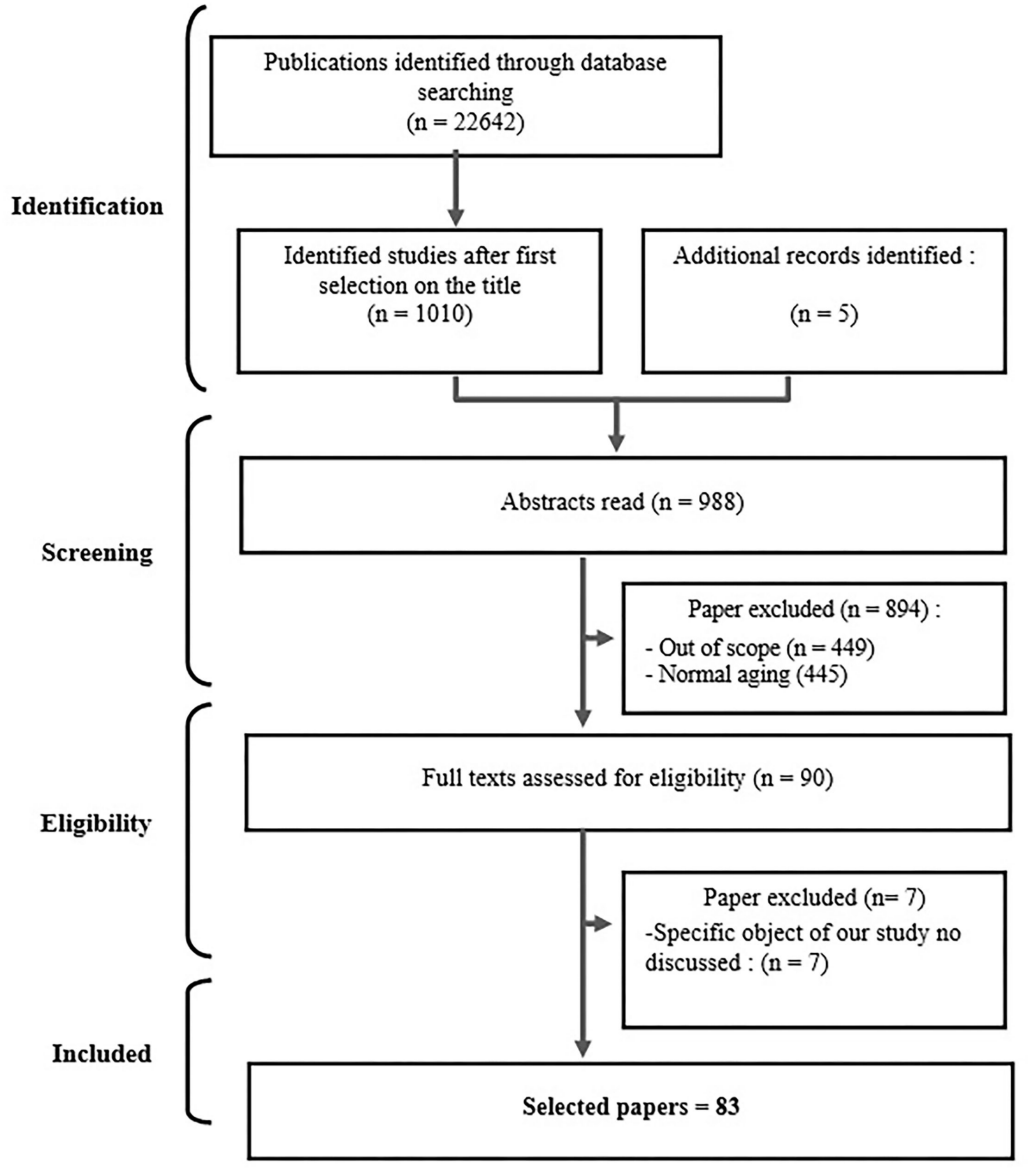
Flow diagram of the selection process.

At the end of this process (Figure 1), 83 articles published between 1981 and 2019 were selected and evaluated (Supplementary Table 1). The limited number of publications providing evidence-based results and the lack of accurate data on the deinstitutionalization of APSDMHD-H led us to rule out a meta-analysis and qualitative systematic review in favor of a narrative review based on a systematic search. Presentation of the results have been performed with the help of the SWiM protocol (5). Presentation of the results mention potential bias isolated in covered studies. To this end, the authors have carried out a thematic analysis (through a theme classification of the different dimensions that appeared in the results of selected studies, using the data extraction table) and grouped them into six distinct categories. Then, a discussion is provided with a list of recommendations to stimulate and guide research practices devoted to aging people with severe and disabling mental health disorders (APSDMHD).

## Results

The selection process resulted in the selection of 83 scientific papers published between 1978 and 2019, mostly from high-income westernized countries with a strong health infrastructure network (Supplementary Table 1). The thematic analysis integrating all the selected productions highlighted six categories. The first reports a lack of research and publications on the process of deinstitutionalization for APSDMHD-H. The second addresses the economic and financial logic associated with the deinstitutionalization of APSDMHD-H. The third focuses on improvement of quality of life and global functioning for deinstitutionalized patients. The fourth category explores the mechanisms of stigma and discrimination associated with APSDMHD. Then, a fifth category reports a lack of community structures dedicated to APSDMHD. Finally, the last category addresses the mechanism of transinstitutionalization and its associated factors.

### A Forgotten Population in Research and Health Policies

While the constant increase in the population of APSDMHD is a major public health issue (14, 16, 21–24), the number of studies on the deinstitutionalization process toward this population (83 articles) seems very limited (8, 14, 25, 26). This paucity of data seems to be primarily related to the population concerned. Also, even though many studies focus on chronic mental health disorders in adults, there is a large body of literature indicating that there are very few clinical, cognitive, epidemiological, and therapeutic studies on APSDMHD (24, 26–31). This, even though this fringe of the population has been showing a steady increase since 1980 and is expected to continue to do so until 2040, particularly in westernized countries such as the United States (21, 32–34).

Concerning the available studies, many authors point out that the existing work does not allow a generalization of the results. Indeed, the published literature often presents certain methodological weaknesses (sample size too small or inclusion of patients with dementia) (27, 30, 31, 35). Similarly, few have proposed an assessment of the factors leading to longer hospital stays for APSDMHD-H (33, 36, 37). In this respect, the clinical evolution of APSDMHD is difficult to evaluate from a psychometric point of view because there are no tools yet available to study the changes in psychological symptoms during aging (16, 35, 37). Moreover, considering age-related predictive variables (emergence of dementia, somatic comorbidities, etc.) to minimize bias is extremely complex to achieve in the context of elderly psychiatry (11, 16, 38, 39). All these variables therefore make it difficult to conduct randomized clinical trials (RCTs) and indeed to obtain generalizable data (11, 30, 38, 39).

At the same time, the issues surrounding APSDMHD seem to be under-explored. For example, WHO consensus statements (2) or the publication of the European commission (40) addressed the issue of cognitive impairment in the elderly, depressive symptoms, or dementia, but not the aging of PSDMHD (11). Moreover, more recent WHO publications (3) still do not seem to have explored this issue further. In addition to their scarcity, work on the existing provision of care for these users seems to focus essentially on the medical approach (14, 26, 41–43). They also neglect the social and medico-social dimensions, which are nonetheless important in supporting APSDMHD, particularly when implementing a policy of deinstitutionalization (14, 26, 41–43). Finally, despite their importance in the daily life of institutions, the difficulties encountered by the managers (medical, social, medico-social, administrative, etc.) of the services devolved to the APSDMHD are rarely mentioned in the literature (9, 29, 31, 34, 38).

Faced with this observation, one of the answers, brought by the literature, seems to be found in the development of gerontopsychiatry (5, 27, 44–46). Unfortunately, this field developed at the interface of several disciplines (gerontology, psychology of aging, neurology, psychiatry, geriatrics) is still in its early stage and has so far provided very little epidemiological and clinical data (27, 30, 34, 47). Recently, a consensus seems to be emerging. It specifies the need to develop more research (epidemiological, clinical, therapeutic, etc.) on APSDMHD, but also more work for the states, institutions and practitioners concerned by this issue (9, 26).

### An Economic Presentation of the Deinstitutionalization Process

Secondly, many articles associate the deinstitutionalization process with an economic logic. It should be remembered that APSDMHD generally have many atypical clinical characteristics, somatic comorbidities and sometimes cognitive impairments, and that the management of these individuals leads to higher costs of care (36, 48). Also, recalling that the proportion of APSDMHD is constantly increasing (28, 49), a mechanical increase in the costs associated with these individuals is estimated by several publications without providing any figures nor accurate data (16, 22, 28, 48–51). Only Bartels et al. suggest that health care support for APSDMHD-H incurs a cost that is two to three times higher than that of their counterparts without severe and disabling mental disorders (21). However, conducting a numerical evaluation to highlight the increased costs incurred by the APSDMHD-H population is quite complex (49, 52). Indeed, studies have difficulty integrating the overall cost of care for this population because the epidemiological databases on which health economics research is based rely on systems only including demographic factors and diagnoses (24).

In this context, some authors affirm that the implementation of deinstitutionalization policies for APSDMHD-H lead to a reduction in costs (17, 39, 49, 53, 54). Only the Canadian study by Reinharz et al. in 2000, focusing on individuals over 50 years of age with severe and disabling mental disorders, was able to shed light on the figures based on a retrospective study carried out between 1989 and 1997 (39). It appears that the average annual cost of care for an individual hospitalized in a long-term care facility was $34,455, while that of an individual who had benefited from a deinstitutionalization process was $31,696. The authors also showed that over the years, this difference had been increasing ($70,109 for hospitalized people and $56,095 for deinstitutionalized people in 1996-1998), suggesting that this increase in costs for APSDMHD-H will continue in the years to come.

Finally, while some authors rely on an economic argument in favor of the deinstitutionalization process, others point out that mental health services for the elderly are generally underfunded in relation to the needs of this population (34, 50, 52–56). In addition, the literature focusing on economic dynamics emphasizes (but is not evidence-based) that despite the proven effectiveness of community-based care for APSDMHD (9), community-based care could reduce unnecessary costs by being more efficient, better organized, identified, and visible (47, 51, 52). This is to limit costly recourse to hospitalization (39), rehospitalization (21, 30, 35), or placement in retirement homes (8, 12, 21, 34). These publications do not however provide precise figures.

### From Stigmatization to the Rejection of Elderly Psychiatric Inpatients From Deinstutionalization Process

A third category of results highlighted the presence of an argument linking the difficulties of the deinstitutionalization process to the phenomenon of stigmatization of APSDMHD. While the stigmatization of older people with mental health problems has been highlighted by many selected studies (8, 22, 25, 42, 44, 57, 58), many authors point out that APSDMHD are subject to a double stigma based on both the stigma of aging and the stigma of mental illness (8, 22, 42, 49, 59). Recently, the systematic review conducted in English and French by Clesse et al. was able to recall that aging individuals with mental disorders are perceived as “unsightly and ugly,” “carriers of difficult and/or violent behavior,” “perceived as a cost to society” and “with diminished or declining cognitive abilities” (8). These representations sometimes lead to the mechanism of self-stigma (8).

Present in the general population, the impact of this double stigmatization would be just as pronounced at the professional level. Thus, some works recall that within the psychiatric field, health professionals adopt a pessimistic and nihilistic stance induced by the idea that this population is “incurable” (22, 42), “unaware of its environment” (55, 59) and by long-term hospitalization (55). Outside the psychiatric field, these individuals are also negatively perceived by certain medical specialties fearing that they will have to manage complex situations correlated to the diagnoses, bed availability/problems and the difficulty of reorienting this population (14, 22, 46). Similarly, some authors recall the difficulties encountered by non-psychiatric institutions by highlighting the resistance of professionals who consider the APSDMHD to be inadequate to these services because of their behavior (34, 38, 60). More recently, a French study of 790 health professionals working with individual carrying psychiatric disorders showed that these professionals had the same representation as the general population (59). These professional attitudes can then lead to the hasty idea that these individuals supported by psychiatry present major adaptation difficulties and lead to professional counter-attitudes (42), apprehension (42), or even the emergence of discrimination in state, institutional and professional support (8, 12, 14, 59).

Finally, the presence of these stigmatizing elements induces the idea that accompanying APSDMHD is too complex or impractical, which often leads to the emergence of institutional resistance (34, 38, 42, 61). On this point, some authors have pointed out that stigma and discrimination mechanisms may have kept APSDMHD away from community support policies (12, 59, 62). Faced with these elements, many state or supranational institutions (2, 3, 63, 64), have recalled that stigmatization mechanisms have deleterious consequences on the organization and delivery of community services, as well as on the living conditions of older people with mental disorders (5, 22, 42, 57, 58). Nevertheless, the complex mechanisms inherent in the diffusion of the dynamics of discrimination within state, institutional and professional policies are still very little studied, and some authors still point out the need to produce more work in this field (8, 59).

### Improvement of Quality of Life and Global Functioning for Deinstitutionalized Patients

Results demonstrated that geriatric psychiatric subpopulation are among the most successfully reoriented in community programs, leading mainly to a reduction of hospital use (60). In comparison to hospitalization, receiving care in the community significantly improves quality of life (particularly in the following dimensions: social skills, recreation and living situation) and life satisfaction (60, 62, 65, 66). It also decreases the yearly hospitalization duration (65), the use of psychopharmacology treatment and the need of case manager supervision (66). Receiving care in community also lead to significant improvements in communication and social contact, autonomy, global functioning, cognitive skills and psychiatric symptoms (60, 62, 65, 67–70). Improvements of participant's psychiatric, social and functional statues appears stable and progressive over time, with the absence of significant deterioration in overall functioning when transferred to community homes (60, 67, 68, 71, 72). Selected studies also demonstrate that APSDMHD express a clear preference for community care (70%) (73), perceived more independence (86%), privacy (93%) (65), and feel more satisfied (72). There is however a negative influence of age on relocation, indicating that younger patients (<70) could benefit more from deinstitutionalization programs (70).

It is as well mentioned that social anxiety symptoms are commonly reported among elderly patients with mental disorders after hospital discharge (71). This is interpreted as a consequence of long-time hospitalization characterized by a reduction in social functioning or communication skills, and the fact that patients in the community need to mainly manage their stress by themselves (71). A decline of global cognition and processing speed is also noticed after a few years in community, but it is mainly due to a genuine decline and aging (70). The most vulnerable patients are at greater risk of injuries than with full hospitalization (60, 67) requiring specific attention from staff.

Finally, it appears that a successful relocation requires careful planning and preparation (participants informed months before relocation, organization of counseling and visits, family members as active participants of the preparation process), an initial 24-h supervision, specific age related medical and psychiatric services, adequate housing supported by government funding, acceptance by local residents, sufficient funds to purchase daily necessities (even if this population is generally below the poverty line) and a routine measure of patient's own attitudes and preferences with detailed patient's problems and needs (65, 66, 71–73).

### A Difficult Community-Based Care Offer and a Difficult Epistemological Identification

One of the remedies to institutionalization of APSDMHD-H is based on the development of community-based services (1, 7, 9, 24, 72, 73). There is however a limited supply of community care and a lack of alternatives to hospitalization (30, 32, 34, 42, 46, 55, 60). Similarly, it would appear that existing services are considered difficult to access and present problems of use (32, 50, 54, 74), by being, in particular, poorly defined and poorly identified (46, 52). Very little data are available in the West (42). For this reason, none of the selected items provide a detailed inventory of existing services and the obstacles encountered. Finally, the lack of innovative structures in terms of social reintegration (7, 27, 30, 60, 75) as well as the lack of specific training on community support for APSDMHD (7, 10, 16, 26, 30, 42, 76), shows that the community care offer for APSDMHD remains to be improved. Where structures are identified, they appear to focus primarily on continuity and coordination of care (46). They may then abandon the psychosocial dimension associated with the deinstitutionalization process (10, 18, 25, 32, 55, 57). There is also evidence that the needs of individuals with psychiatric support in terms of recreation, culture or social connection are poorly addressed (10, 26, 37, 42, 75, 77). Some publications also denounce the mechanism of ghettoization of PSDMHD in poor and disadvantaged neighborhoods (55, 56).

Finally, it seems that the support needs of APSDMHD are still difficult to assess because of the difficult overlap between mental health and geriatrics (32, 46, 78), leading to both specific multi-professional care (46) and improved coordination between services (42, 74, 75). However, the confusion still existing between gerontopsychiatry and psychogeriatry (16, 44, 78, 79) as well as the difficulties existing between psychiatry and gerontopsychiatry teams (44, 79) show that community support for APSDMHD remains very difficult in Europe (9, 16, 47) and in the USA (76). In the end, all these studies point out that the process of deinstitutionalization of the APSDMHD cannot be fully realized due to the lack of numerous and efficient community alternatives.

### From a Lack of Community Services to the Phenomenon of Transinstitutionalization

During our categorization, 37 articles indicated the emergence of a phenomenon related to the mechanism of deinstitutionalization of APSDMHD-H: the phenomenon of transinstitutionalization. Favored by the lack of available psychiatric hospital beds and the decrease in length of stay (42, 53, 75, 80, 81), the phenomenon of transinstitutionalization describes the migratory process by which a patient is transferred from one institution to another (52). The greatest paradox of the deinstitutionalization movement initially dedicated to APSDMHD-H has been the transfer to the sector concerned by aging (retirement homes, etc.) of the support for APSDMHD initially cared for in hospital (24, 34, 49, 54, 74, 75, 80–82). This, when many of these individuals could have benefited from community support.

A significant body of literature has highlighted the fact that APSDMHD-H have benefited very little from the deinstitutionalization movement (32, 34, 38, 42, 74, 83, 84). Again, there is few available quantitative data on this point. The work of Kermis (32) and Talbott (49) show that in the late 1980s in the USA, only 25% of APSDMHD lived in the community. In 2005, Grabowski et al. (56) estimated that the number of APSDMHD living in psychiatric hospitals was 54%. More recently and in France, this trend was confirmed in 2011 with only 7% of the APSDMHD lived in independent housing (84). The latter is in line with Jovelet's estimate (34) that only 13% of APSDMHD-H leave the psychiatric hospital in favor of inclusion in the community. Finally, these figures are also linked to 2009 data recalling that 44% of French APSDMHD were living in psychiatric hospitals (85).

Typically, in many westernized countries the address of the transinstitutionalization dynamic is the retirement home (16, 30, 34, 38, 49, 54, 75, 82, 83). As early as the 1980s, this movement was already denounced by Goldman et al. (78), Kermis (32), and Freiman et al. (52), for whom 30% of APSDMHD-H were directly referred to retirement homes. Today, current statistical data show that this dynamic is still at work (74). Thus, in the USA, between 2009 and 2011, 500,000 APSDMHD resided in institutions dedicated to aging (retirement home etc.) (54, 56). Similarly, in France, between 2011 and 2018 this figure was 40,000 or 28% of the population (34, 84). At the dawn of its 60 years of existence, some authors therefore consider that the deinstitutionalization movement has not proven to be efficient for APSDMHD (74, 81). Others view retirement homes today as new mental health care homes (34, 42, 54, 56, 75).

In parallel, scientific literature has identified some of the factors that have promoted and driven the transinstitutionalization mechanism for APSDMHD-H. The first factor is based on the preconceived idea that aging can lead to premature loss of autonomy in individuals suffering from psychosis (30, 34, 42, 52). Another factor is based on the fact that the association of psychiatric, cognitive and somatic pathologies implies a strong coordination of care that is difficult to achieve within a community-based approach (28, 30, 33, 34, 55, 86). A third factor seems to be related to the lack of effective therapies for APSDMHD, given that most of the care offered to this population remains focused on medication (18, 26, 53, 82, 87). In the absence of clinical studies, drug therapy is then routinely described as inappropriate (29). This is even though some authors point out that, taking into account the pharmacokinetic data and the risk of drug iatrogeny, doses of psychotropic drugs are progressively limited with advancing age (28, 29, 33, 34). A fourth factor is related to the economic interest of these guidelines, which are considered less costly for the state (34, 52, 56, 75). Similarly, referral may be facilitated by a funding system (less favorable to the problem of aging than to psychiatric problems) that is more advantageous for the states (16). Today, these elements tend to be reinforced by certain economic policies aimed at limiting the crisis of older age institutions in the face of the heterogeneity of the publics they cater for (30, 56).

Despite the increase in financial resources made by some countries, these are considered insufficient (15, 56, 75). Thus, the provision of these structures with staff and activities for their residents is often perceived as insufficient (30, 37, 42, 76, 81). Likewise, professionals do not seem to be really trained to accompany aging psychological problems (30, 34, 38, 42, 87). As a result, these institutions struggle to provide the necessary psychological care (24, 32). In the end, this dynamic could lead to an accentuated degradation of the residents accommodated (32, 34). All these elements are now considered inherent factors in the process of transinstitutionalization of APSDMHD.

## Discussion

The study of the existing links between the deinstitutionalization mechanism and the APSDMHD-H identified six categories: “the lack of publication and work on the deinstitutionalization of APSDMHD”; “the association of the deinstitutionalization mechanism with an economic logic”; “the improvement of quality of life and global functioning for deinstitutionalized patients”; “the presence of a strong stigmatization of this population”; “the lack of community structures for these individuals”; and “the emergence of a dynamic of transinstitutionalization.”

The problem addressed in this article appears to be only dealt with by high-income westernized countries. This particularity can be explained by the cultural influence and history of these countries. While the management of insanity and aging is for most worldwide countries a family affair (86), the modalities of family life in Anglo-Saxon countries such as those of Western Europe are today more oriented toward a nuclear model of the family, which effectively excludes the elderly (88, 89). As a result, aging individuals in highly westernized countries have been massively subjected to a dynamic of institutionalization (9). Likewise, since the 19th century, only westernized and wealthy countries had developed methods of accompanying madness through asylum (90). The management of aging and psychological pathology by westernized countries has therefore been achieved through policies of institutionalization of these individuals. It is therefore possible to consider that the movement to deinstitutionalize APSDMHD-H is directly related to two factors: the cultural factor that transferred the responsibility for aging to the states and the failure of health policies aimed at institutionalizing insanity in western states. In addition to the combination of these two factors, the western origin of the publications could also be explained by the fact that high income countries receive more research funding.

Also, we noted that it is possible to categorized all the selected publications into three time periods. A first group of publications corresponds to the studies promoted during the 1980s in the countries that initiated the concept of deinstitutionalization (32). These publications showed that the most autonomous APSDMHD-H could leave the hospital, while pointing out the difficulties of society in including them and ensuring their support (24). A second period of publication between 1990 and 2005 seems to be characterized by a scarcity of literature and a focus on the economic logic associated with the deinstitutionalization of APSDMHD-H. Finally, the last publication period (2005-2019) notes the failure of deinstitutionalization policies toward APSDMHD-H while isolating the potential factors linked to this failure (stigmatization, territorial disparities, lack of community resources, transinstitutionalization…). This division could be explained by the fact that during the 1980s, a first wave of deinstitutionalization may have concerned all autonomous individuals, resulting in the treatment of the problems of APSDMHD-H at the margin without any real evidence. At that time, deinstitutionalization was more aimed at social reintegration (55, 67). Since the emergence of social rehabilitation that allows for effective psychosocial rehabilitation work cannot be relied upon, it is possible that many individuals were not considered by the first wave of deinstitutionalization at the time. Secondly, the generalization of deinstitutionalization policies in the West during the years 1990-2005 led countries to generate a second phase of deinstitutionalization. Associated with the growing influence of nosographic classifications, the latter was based on a categorization of the psychiatric population by psychic disorder (schizophrenia, bipolarity, etc.). This process is justified by the specificities of each pathology and the cognitive mechanisms associated with it (e.g., the difficulties in TOM in schizophrenic individuals) and has probably directed the attention of institutions toward the most studied categories of individuals (such as the onset of schizophrenia). As a result, it was able to mask the problem posed by the population subset made up of APSDMHD-H. Finally, when the first two waves of deinstitutionalization were completed, many institutions were able to note that one of the remaining major population groups was APSDMHD. This clinical finding may have led to more recent publications on the challenges surrounding the deinstitutionalization of APSDMHD-H.

In terms of the categories in the results section, the first noted the lack of literature on the deinstitutionalization of APSDMHD-H, but also the lack of epidemiological and clinical studies on this population. First, the organization of the deinstitutionalization movement into three distinct waves could explain the recent interest of research in this issue. Similarly, it is possible that the average increase in life expectancy (28, 33, 34) including in the psychiatric population (13) the dissemination of public health policies aimed at reducing the impact of somatic comorbidities (78), the emergence of new generation antipsychotics (29, 35), improved diagnostic capacity to differentiate neurological disorders from psychiatric pathologies (91) and improved geriatric care (28, 58, 79) have led to an increase in the life expectancy of APSDMHD (15, 28–30, 47). Whereas previously a lower life expectancy did not allow them to be considered as a population, it is possible that the increase in life expectancy of APSDMHD may have created a mechanical increase in the number of APSDMHD-H. This increase, which would need to be assessed through epidemiological studies, could then explain the scarcity of publications on this population and the very recent emergence of research issues related to the deinstitutionalization of APSDMHD-H.

We then highlight the links between the deinstitutionalization of APSDMHD-H and economic logic. Here, we were able to highlight the difficulty of studies to promote evidence assessing the full range of subsidiary costs caused by the iatrogenic consequences of the institution (increased tobacco consumption, obesity, increased loss of cognitive skills, etc.). Similarly, the costs associated with setting up a community support dynamic (support time, influence of territorial disparities, team coordination time, etc.) are rarely evaluated. In addition, it can be noted that economic logic is often presented as an argument in favor of implementing deinstitutionalization policies for APSDMHD-H. However, engaging in a policy of deinstitutionalization based mainly on the economic factor is ethically questionable. By not linking the deinstitutionalization mechanism with the desirable community approach and the expected quality of life gains, many institutions could then confuse deinstitutionalization with de-hospitalization (92). As a result, they could force many APSDMHD-H into a violent and unadjusted institutional process aimed solely at reducing psychiatric costs/ beds. Territorial work aimed at strengthening community support arrangements is therefore desirable upstream of a deinstitutionalization policy, bringing the notion of economy suggested by the deinstitutionalization of APSDMHD-H to the forefront.

The third category highlights the positive representation of deinstitutionalization for elderly patients also as the benefits of this relocation for autonomy, privacy, psychiatric symptoms, social skills and communication. However, a successful relocation need to be fully prepare months before the event, with counseling, visits and supervision. Moreover, a successful relocation requires a stable financial situation for the patient, as well as specific geriatric medical and psychiatric cares, and better specialization of structures and employees to prevent risks of deinstitutionalization after a long-time placement. These specificities are prominent facilitators of a successful relocation and should be applied in all institutions.

In the fourth category, much has been written about the links between the stigmatization of APSDMHD and the emergence of discrimination against this population. However, many of the results presented by the authors come from extrapolation from studies on the association of “mental disorders” and “aging.” For greater precision, it would be very useful to conduct cohort studies specifically on the mechanisms of stigmatization of the aging psychiatric population with severe and disabling disorders. Similarly, it would be relevant to extend the work on referral mechanisms for APSDMHD-H for alternatives to community hospitalization to identify the brakes and levers to be activated in order to propose equitable and ethical public policies (59, 93). In the long term, the literature could provide a more accurate model of the influence of the mechanics of stigmatization on discrimination against APSDMHD-H.

Then lack of community structures for APSDMHD showed that despite the development of ambulatory structures, these sometimes do not seem specifically adapted to receiving APSDMHD. It seems that professionals trained in gerontopsychiatry are the most likely to carry out this work at the interface of several disciplines (16, 76). The construction of community initiatives that fully integrate APSDMHD into territorial projects would benefit from being carried out and then disseminated to the scientific community. In doing so, it would show how to overcome the territorial challenges and the difficult coordination necessary for the balance and quality of life of the APSDMHD within the community.

Finally, the observation that the deinstitutionalization/de hospitalization process has led to the emergence of a transinstitutionalization process for the elderly shows that the process of deinstitutionalization of APSDMHD-H has failed. While these individuals can live within the social fabric (83) by receiving support tailored to their problems, the transinstitutionalization mechanism reflects the lack of accessible community structures, as well as the almost systematic exclusion of APSDMHD from existing structures when these situations are presented. In the same way, the transinstitutionalization mechanism also questions the modalities of guidance for APSDMHD-H and carried by specific hospital institutions. To finish, the transinstitutionalization mechanism is a reminder that a significant fringe of APSDMHD now live in institutions for the elderly. Under these conditions, particular attention must be paid to accompanying the residents of these institutions, but also support and training for these professionals seems necessary.

### What Are the Prospects for Research and Clinical Practice With APSDMHD?

The findings of our literature review allow us to make a few recommendations for researchers and professionals concerned with APSDMHD. First, it seems essential to encourage the conduct of epidemiological studies on APSDMHD and APSDMHD-H (16, 29, 76). As well, pharmacological studies that go beyond symptom reduction by questioning the recovery process would be very useful (14). Then, recalling that adapted cognitive and psychoaffective evaluations for this population are recently developed (94) while cognitive remediation and psychosocial rehabilitation programs allow significant cognitive and social gains, these latters would benefit from developing a specific adaptation to the problems of APSDMHD (5, 25, 77). Similarly, longitudinal studies based on homogeneous cohorts evaluated using valid and standardized tools should also be encouraged (30).

Secondly, the recognition of research work on APSDMHD would make it possible to federate the scientific community within the gerontopsychiatric stream and facilitate epistemological identification, which is still difficult (16, 47, 76). This perspective would allow the emergence of an identifiable theoretical trend that would ultimately facilitate training for students (76) and professionals (22, 46, 77).

Third, for each country, an inventory of existing community structures that includes a possibility of support for APSDMHD would allow for an effective evaluation of existing networks (associating psychiatry, geriatrics, and aging professionals), territorial disparities and the needs of this population (24, 28, 47, 58).

Fourth, studies on the mechanisms of stigmatization of APSDMHD and the institutional consequences of stigmatization would provide useful insights. The latter could potentially identify the barriers that caused the transinstitutionalization mechanism. In doing so, it would then be possible to develop appropriate training dynamics based on scientific advances in the field of APSDMHD, concerned about their well-being and guaranteeing equity in access to care.

### Strength and Limits

One of the main strengths of this work is that it is the first to focus the body of literature on the deinstitutionalization of APSDMHD-H. Similarly, the association of a second language (French) has shed more light on the issue. Also, the systematic used methodology let us to gather all existing publications on this topic and provide an historical perspective. On the other hand, due to a lack of specific information on this topic, it has not been possible to point potential variations between geographical area regarding the deinstitutionalization of APSDMHD. Finally, the small number of writings, and paucity of qualitative and quantitative data did not allow for a selection based on methodology. This review then integrates a variety of writings including clinical and economic studies, but also some positions of clinical authors and literature reviews.

## Conclusion

While the spread of de-institutionalization policies has led to a change in the support of mental illness in many countries, this movement has not really had an impact on aging people with severe and disabling mental disorders. The combination of a lack of studies on this population, a lack of community services for this population, and strong stigmatization mechanisms has led to the exclusion of APSDMHD-H from community health care policies or, failing that, to a massive shift toward transinstitutionalization. However, those benefiting from this movement present a better autonomy and communication, an amelioration of their social skills, a better sense of privacy and a reduction of the psychiatric symptoms. The development of studies in the field of gerontopsychiatry could make it possible to encourage the development of ethical institutional reflections that are clinically adjusted to the specific clinical situation of these individuals.

## Author Contributions

SS and CC conceptualized and designed the study. SS, CC, and AJ conducted the selection process. SS and AJ interpreted the data. SS and CC wrote the paper with the contribution of AJ. MB oversaw the data analysis and interpretation, helped in the selection article, and contributed to the writing article. All authors have read and approved the manuscript.

## Conflict of Interest

The authors declare that the research was conducted in the absence of any commercial or financial relationships that could be construed as a potential conflict of interest.

## Publisher's Note

All claims expressed in this article are solely those of the authors and do not necessarily represent those of their affiliated organizations, or those of the publisher, the editors and the reviewers. Any product that may be evaluated in this article, or claim that may be made by its manufacturer, is not guaranteed or endorsed by the publisher.

## Abbreviations

APSDMHD: aging people with severe and disabling mental health disorders
APSDMHD-H: aging people with severe and disabling mental health disorders-hospitalized
PRISMA: preferred reported for systematic reviews and meta-analyses
PSDMHD: people with severe and disabling mental health disorders
TOM: theory of mind
WHO: world health organization.
