# Supplementary material for: Process of Deinstitutionalization of Aging Individuals With Severe and Disabling Mental Disorders: A Review
Source: Front Psychiatry. 2022 Mar 24;13:813338. doi: 10.3389/fpsyt.2022.813338 (PMC8987193; doi:10.3389/fpsyt.2022.813338)
Supplement: Supplementary file 1 [file Table_1.doc]

Table 1 : Study assessment

| Authors | Year | Article | Method | Results |
| --- | --- | --- | --- | --- |
| Altamura, C., & Elliott, T. | 2003 | Literature review | This article highlights why schizophrenia in the elderly is a special case requiring special care and suggests treatment approaches for this population. | In patients over 65 years of age, atypical antipsychotics are a better alternative. The use of these new agents allied with community mental health and care from nurses can be a powerful combination in response to the unique requirements of this population. |
| Amerongen, A.P. | 2009 | Opinion article | The authors approach actual social and medical reality to consider how to best optimize providing care for old schizophrenic people. They include proposals expressed by the family circle. | - The management of old and sick people must be organized by a multidisciplinary team.  - Teams must cooperate with decision-makers and the elective representative.  - Suitable structures need to be created.  - The dual discrimination/stigmatization issue needs to be combated. |
| Aro, S., Noro, A., and al. | 1997 | Longitudinal study | The success of the Finnish deinstitutionalization policy for people over 65 years of age between 1981-1991 was estimated. The staff of 21 districts filled in a form indicating the number of hospitalization days, the diagnosis, and the patient autonomy level. A days of hospitalization/number of patients ratio compared with the total population enabled the authors to prepare standardized results. | During the period of study, the average duration of stay was shortened for elderly people in institution but increased in the mental health services.  Patients from psychiatric hospitals continued to remain institutionalized over this 10-year period. |
| Bartels, S.J.,and al., | 1997 | Retrospective cohort study | Investigation about elderly people's needs related to medical health services sponsored by the legislative Assembly of New Hampshire State and the Division of Mental Health. Data from 2046 patients over 60 years old were collected regarding mental health in New Hampshire based on 10 mental health centers at the end of 1994.  Subjects were classified into 3 groups: schizo-affective disorder (N = 55), bipolar disorder (N = 39), or major depression (N = 90). | Authors noted that the elderly people using the least mental health services are those suffering from affective disorders, anxiety and organic mental disorders.  Next came "most patients with schizophrenia living in supported residences compared to less than one-quarter for patients with depression and about one third of the patients with bipolar disorder".  The difference of psychiatric symptoms between the 3 groups was measured by a multivariate analysis of variance (MANOVA) and "the multivariate F for this test was significant, F(6, 360) = 18.39, p < .001, indicating significant differences in symptoms across the diagnostic groups" (p.52). |
| Bartels, S.J., & Pratt, S.I. | 2009 | Literature review | This selective review considers literature published in peer-reviewed journals over the last 18 months on the psychosocial functioning of elderly people with a mental disorder. | Social isolation, depression, cognitive disorder and chronic somatic disease are thought to be responsible for a low quality of life and functional level for that population. Psychosocial readjustment, cognitive remediation and readjustment support are efficient ways of improving how this population functions. |
| Berry, K., & Barrowclough, C. | 2009 | Literature review | Literature review in English language on 7 bases of specialized data aimed at establishing the psychosocial needs of elderly patients with schizophrenia. | Elderly patients with schizophrenia share some identical needs with younger patients. These especially include understanding and managing symptoms, cognitive and social functioning, psychological resilience, social support and life quality support, physical health, and access to treatment. The care offer must be mutualized. |
| Biering, P. | 2019 | Literature review | Databases from 2000 to July 2017 were searched to find the most useful health care approaches for seniors with mental health issues. A critical interpretative synthesis was used to analyze and interpret the results. | Few elderly people have access to psychotherapy. Holistic or integrated models of health care have emerged in recent years. These models focus on physical and psychosocial well-being and have shown promising results. |
| Blank, K., and al. | 2005 | Retrospective cohort study | Demographic, clinical and treatment data on 384 patients were gathered from 464 hospitals between July 1^st^ 2000 and June 30th 2001. A model of linear regression was developed to measure the contributions of each independent variable explaining variance in the duration of continuous stays. | The eldest patients suffering from serious mental disease often longer hospitalization than younger adults.  No social factors were found to explain differences in the duration of hospitalization between young and older subjects.  The health coverage system does not have any impact on the duration of stay. |
| Carling, P.J. | 1981 | Opinion article | Through a critical review of the work of Shadish and Bootzin (1981), the author defines the broader context of the national problem of nursing homes. It summarizes the most glaring historical shortcomings in the use of the institutions at the heart of a mental health system. | This presentation raises serious questions about Shadish and Bootzin's proposal to use nursing homes as the basis on which to rebuild an improved system of community services for people with chronic mental illnesses. |
| Charazac, P. | 2011 | Opinion article | The author takes his own experiences on the field as a basis to question the relevance of an age criterion in geronto-psychiatry. | The future of geronto-psychiatry will depend on the way psychiatrists address the issue of the adequacy of the mission and the resources available and how these work in harmony other devices for aging psychotic subjects. |
| Clesse, C., and al. | 2016(a) | Literature review with systematic strategy | Exploration of 14 specialized databases in French and English aimed at illustrating the factors which facilitate the orientation of ageing subjects with a psychiatric pathology. | -Alternatives to hospitalization are today proposed to subjects with psychic disability but ageing subjects do not have access to these.  -Psychosocial rehabilitation methods are today described as the most efficient but are not proposed to elderly people.  -This population is affected by age-related stigma. Chronologic age could be replaced by a "psychosocial age". |
| Clesse, C., and al. | 2016(b) | Literature review | Data found from a selective work on 10 specialized data bases were gathered, categorized and described to bring out the impact of discrimination on the ageing population with psychiatric pathology. | The results highlight three main categories of social representations indicating that ageing and mental pathology are sources of stigma and discrimination. The latter retract this population's deinstitutionalization process. |
| Cohen, L., and al. | 2014 | Opinion article | "State of the elderly Psychiatry in France related to national and international data" (p. 1) | A demographic evolution is facing psychiatrists working with ageing patients whose clinical particularities require special training for care staff and a specific care offer. Psychiatry for the Elderly must develop a dynamic that structures and makes the care offer readable, dedicated and organized. |
| Cohen, C. I., and al. | 2008 | Literature review | "The aim of this column is to review recent findings on the outcome and associated features of clinical symptom and social well-being categories for older adults with schizophrenia" (p. 232) | Half of ageing patients with early-onset schizophrenia see their general functioning improve as they get older. Research must continue into longitudinal studies to reinforce the general implementation of these results. |
| Cohen, C. I., & GAP Committee on Aging. | 2000 | Summary of a GAP committee report | Report focused on the schizophrenic population and getting old with this disorder. | The committee makes several recommendations "improving the illness-related behavior of older persons with schizophrenia and their service use", "enhancing services for older persons with schizophrenia and their caregivers" and "improving the knowledge base and service delivery of psychiatrists and primary care physicians serving this population" (p.301) |
| Dallaire, B.,and al. | 2010 | Qualitative study | Between 2007 and 2008, an exploratory research approach based on qualitative methods was conducted. Data was collected in establishments and organizations in the Quebec city region. Exploratory interviews were carried out with community and institutional stakeholders practicing psychosocial activities with psychotic ageing people. The 23 exploratory interviews were the subject of a thematic content analysis with the NVivo software. | Ageing people with psychiatric pathology live a life marked by a lack of support, resources and power. They go from one institution to another and need a particular form support. These paradigms are thought to be linked to mental illness and long-term psychiatric institutionalization. |
| Davtian, H. & Scelles, R. | 2013 | Opinion article | Putting the status of the caregiver into perspective through the history of the evolution of the family’s role in psychic care. | The prism of the caregiver does not enable to take into consideration all the issues for a family living with schizophrenia on a daily basis. |
| Dumand, I., and al. | 2018 | Literature review | Two literature reviews were carried out for this article on the calculation of a psychosocial age: one onto the social representations of seniors with a psychiatric disorder and the second into the impact of those representations on the accompaniment of these persons. These two reviews were conducted from 9 databases of French and English articles published between 2000 and 2017 | The "psychosocial age" tool enables professional pluri teams to assess the potential for a real mobilization of psychotic subjects - particularly seniors - and enables the stigma to which this population is subjected to be combated. |
| English, J.T., and al. | 1986 | Observation study | The working group procured a large abstract data set on patients who had been discharged from acute care general hospitals during 1980-1984; the data were derived from the Uniform Hospital Discharge Data Set. Discharge records with a primary or secondary psychiatric diagnosis as identified by ICD-9-CM codes were obtained from 1195 acute care general hospitals - 20% of all U.S. general hospitals. | Health Care Financing impacts the path of support for elderly who remain institutionalized in great numbers.  “Table 2 compares the coefficients of variation in the APA data set and those from the Health Care Financing Administration data derived from the 1981 file of the Medicare Provider Analysis and Review (MEDPAR). Of the nine psychiatric patient DRGs, none had a coefficient of variation less than 0.94, and 55% had coefficients greater than 1.00; that is, the variability was greater than 100%.” (p 134) |
| European Commission. | 2005 | European action plan | Authors are taking stock of the situation, bring many elements to launch a European debate and develop one or more strategies for action. | The main priority is to make care services available and provide effective management of quality mental health. The actions implemented to do so are explained: on one hand, the dissemination of knowledge and prevention of certain behavior caused by mental health in children and young adults and on the other hand, promoting the inclusion of people living with mental illness. |
| Fisher, W. H., Geller, J. L., Pearsall, D. T., Simon, L. J., & Wirth-Cauchon, J. L. | 1991 | Quantitative study | Case mix comparisons through 3 types of data obtained from Massachusetts Department of Mental Health Monthly Facility Reports. These data contain information on patient demographic and diagnostic characteristics as well as prior hospitalization and length of stay. | The development of a continuum of community-based care for chronically mentally ill elderly patients can all but eliminate the need for state hospital care across the full spectrum of psychogeriatric patients.  The geriatric subpopulation are among the most excluded group from national state hospital censuses and represent a group for which significant return on investment in community programs can be achieved, at least in terms of reducing the use of the state hospital.  People receiving care in the community enjoy a superior quality of life to that experienced by comparable state hospital patients. However the most vulnerable patients are at greater risk of injuries than with full hospitalization.  Of the 43 patients discharged to community settings, only four (11%) were ever re-hospitalized, three individuals once and one individual twice.  Even structures serving substantial numbers of psychogeriatric patients often have no specialty staff and few specialized services for this population. The development of innovative alternatives to the state hospital for this population would be welcome. |
| Fisher, W.H., & Moak, G.S. | 1990 | Observational study | In November 1987, the authors carried out an investigation by questionnaire in 284 psychiatric hospitals to observe the functioning of geriatric services. | Despite "major trans-institutionalization" in the psychiatric sector, the elderly can still be counted among the census of American state hospitals. In 1984, people over 65 accounted for 20% of the country's public hospital census.  Unlike their counterparts 30 years ago, many were hospitalized more for residential care than for psychiatric care. |
| Freiman, M.P., and al. | 1990 | Observational study | “This article explores the national implications of the provisions of the OBRA 1987 reform of nursing homes for the mentally ill” (p. 49). | OBRA 1987 requires an elaborate selection process to determine who among the mentally ill should be kept in nursing homes. It is hard to find adequate alternatives for those with psychiatric problems who are inappropriately placed in a nursing home. A lot are sent to acute and long-term care units, retirement and nursing homes, hotels or end up on the streets. |
| Frémont, P. | 2004 | Opinion article | From the recent psychiatry history and evolution of the mental pathology, a question about the fate of ageing schizophrenic subjects is being raised. | The expected significant aging of the population must lead to questions about patients with chronic psychiatric pathologies and especially schizophrenia. Research is required into this question. |
| Furlan, P., Zuffranieri, M., Stanga, F., Ostacoli, L., Patta, J., & Picci, R. | 2009 | Longitudinal study | The longitudinal study focused on the final patients (176 participants) discharged from the psychiatric hospitals of Collegno and Grugliasco. Seven assessments over a four-year period. These periodic assessments enabled evaluation of overall trends in the resettling process. Data were collected by the medical team (which included some of the authors) that oversaw the transition process in the Turin area, chiefly by setting goals and monitoring patients’ clinical and functional outcomes. | Measures at final state of the process:  Total scores on the BPRS and on all five BPRS subscales indicated improvement, although to differing extents. The most marked improvements in subscale scores were for thought disturbance (effect size=.26) and anxiety-depression (effect size=.22).  Slight improvements were observed in communication, autonomy in activities of daily living, and cognitive skills. Although the improvements were statistically significant, in concrete terms they would appear to indicate stability of these skills over time.  The score for social contact indicated a more marked improvement (effect size=.26).  An improvement was evident on both subscales—volition and aggression—but only for volition was the effect size (.26) significant.  The results indicate that discharge led to a positive general trend, as evaluated by clinical and functional scales. The improvement appeared to be stable and progressive over time, although obviously a patient’s age may have influenced this trend. Indeed, preconceptions of some psychiatric hospital staff about the chronicity of illness of these patients might have led them to treat some long-stay patients as geriatric rather than psychiatric cases. |
| Gakou, S., and al., | 2019 | Observational study | Through field experimentation, authors observed the relevance of integrating a nurse with advanced practice skills to the psychiatric team with the aim of empowering the elderly with psychiatric disorders so that they can remain in their homes. | The nurse working as the mission head with advanced practice skills can respond to problems encountered by ageing subjects displaying a psychic disorder which are otherwise not taken into account. (p.111), However, this job does not exist in France. |
| Gerber, G. J., Coleman, G. E., Johnston, L., & Lafave, H. G. | 1994 | Longitudinal study | "Clients discharged from a psychiatric rehabilitation program at Brockville Psychiatric Hospital (BPH), Ontario, Canada were interviewed in 1988 and again in 1990. The results of the first interviews were previously reported.” The clients had been hospitalized at BPH for an average of 48 months. Following attempts to contact the 55 people interviewed in 1988, 43 of these participated in a second interview in 1990. Most clients were receiving little follow-up care from BPH. | 43% of patients had been re-hospitalized during the 3 years following the deinstitutionalization. However, the duration of hospitalization during the year decreased by 43% between 1988 and 1990, from a mean of 105.8 days in 1988 to a mean of 60.4 days in 1990.  Patients showed better scores than during hospitalization at 3 specific dimensions of quality of life: social skills, recreation and living situation.  86% of patients reported that life outside the hospital offered more independence, 93% felt it offered more privacy after 3 years of deinstitutionalization (77% in both measures for first year).  There was no increase of inappropriate behaviors.  The favorable outcomes for people discharged from psychiatric hospital in the current study seem to depend on several factors. All clients interviewed had adequate housing supported by government funding. Housing was located in areas where clients appeared to be accepted by local residents. Although all clients were living below the poverty line, they had sufficient funds to purchase daily necessities. |
| Goldman, H.H. | 1986 | Observational study | New analysis of data from the 1977 National Nursing Home Survey, including data not available earlier, estimated that 668,000 patients with chronic mental illness reside in nursing homes. | Residents with mental health problems were more likely to have behavioral problems and stay in institutions for much longer. Data illustrates the wide range of needs of residents of mental health care homes and reinforces the importance of assessing and improving the relevance of care provided |
| Grabowski, D.C., et al. | 2010 | Literature review | Using the Donabedian frame, the authors estimate the quality of care provided to ageing with mental disease and reside in nursing homes. | The authors found "a high prevalence of individuals with a mental illness other than dementia are present in nursing homes" (p.645), that their treatments are often of bad quality and that this is linked to factors such as resident well-being, provider standards and financial considerations. |
| Graham, N., and al. | 2003 | Technical consensus declaration | Joint production of the Old Age Psychiatry Section of the World Psychiatric Association and World Health Organization, in collaboration with several NGOs and experts from different regions, to describe the nature, cause and consequences of stigma of the ageing population with mental pathology. | Proposal for tools for the debate around stigma of the ageing with mental disorders. |
| Hanon, C., & Camus, V. | 2010 | Opinion article | The status of psychiatric training regarding the elderly in Europe and worldwide. | Improvement of knowledge and know-how in the field of diagnosis and management of psychiatric elderly disorders has become a widely recognized necessity. Programs must be developed and specific care services to improve the care offer for this population. |
| Hobbs, C., Newton, L., Tennant, C., Rosen, A., & Tribe, K. | 2002 | Longitudinal study | Quantitative evaluation was conducted using the Brief Psychiatric Rating Scale, Life Skills Profile, Social Behavior Scale, Montgomery Asberg Depression Rating Scale and Quality Of Life measures. Assessments were completed prior to discharge and at two- and 6-year intervals following community transfer. Repeated measure analysis was utilized to determine changes in outcome variables over time. The residents’ perception of 6-years of community living was explored in qualitative semi-structured interviews. Details of accommodation, level of care, readmissions, incidents and medication were also documented. | The major finding of this study was that residents had improved life satisfaction living in the community. Their clinical stability over 6 years of community life was maintained in the face of a significant decrease in both the overall level of psychopharmacology and in the level of case manager supervision.  The residents maintained community tenure with significant improvement in quality of life and a reduction in medication, supported by a mental health system with adequate community resources.  Issues regarding continuing rehabilitation and social integration need to be addressed.  Further deinstitutionalization will require 24-h supervision for most initially and for some on a continuing basis. An ageing population will require specific age related medical and psychiatric services (age care hostels, clustered housing models…) |
| Hogg, L. I., & Brooks, N. | 1990 | Longitudinal study | A group of 24 "new chronic" schizophrenic inpatients was compared with a group of 19 day-patients matched for duration of current care; both had spent between 1 and 6 years in continuous inpatient or day-patient care.  The groups were compared on a variety of clinical, demographic, and social variables to identify specific variables that might distinguish the new chronic inpatient group. | The demographic characteristics of the groups were remarkably uniform on most variables including mental states, social and family support, deviant behavior scores.  The majority of both groups expressed a clear preference for community care (70%).  In clinical practice, it is imperative that decisions regarding a patient’s future care be made in the context of a comprehensive and detailed objective assessment of that patient’s problems and needs.  Results - a measure of patients’ own attitudes and preferences should be routinely included in any detailed patient assessment and be considered in relation to decisions about their future care. Patients in the community do not necessarily have more social contacts than inpatients, suggesting the need to look more carefully at ways of facilitating patients’ integration into the community in which they live.  The results therefore highlight the urgent need for more comprehensive and detailed assessment of patients in relation to decisions about retention in or discharge from inpatient hospital care and also the need to identify objective predictors of the success of such decisions. |
| Houbin, B. | 2015 | Observational study, retrospective | Description of the context of the creation of the elderly psychiatrist team West 94 (EMPPA, France, West 94) of the hospital group Paul-Guiraud de Villejuif, whose mission is to evaluate and take decisions about patients over 60 with psychiatric disorders. | This device facilitates evaluation and care access for elderly patients with psychiatric disorders thanks to home interventions and an appropriate use of the territory’s resources. The network at the interface of psychiatry, somatic and social addresses certain issues related to demographic change. |
| Jalenques, P. | 2009 | Review article | -Prevalence or incidence studies in the elderly population  -Long-term follow-up studies in patients with early-onset schizophrenia.  -Higher clinical tables encountered in patients with schizophrenia advanced age.  -Differential diagnosis  -Way of life analysis, family and social about psychotic old subjects. | The number of ageing patients with schizophrenia is on the rise. It is important and urgent to improve our knowledge to help effectively manage these patients by integrating the somatic and psychiatric sides of the disorder with clinical and therapeutic goals taking into account their care and care pathways. |
| Janocko, K. M., & Lee, S. S. | 1988 | Opinion article | Authors discuss "the ethical issues faced by psychologists working as members of an interdisciplinary team in a psychiatric hospital in light of the current trend and policy to deinstitutionalize" (p. 522) | Deinstitutionalization decisions about ageing psychotic patients must be taken on a case-by-case basis. A great deal of change is required at the care system level and in the political system to ensure the ethics of aging patient care in a deinstitutionalization context. |
| Kumazaki, H., Kobayashi, H., Niimura, H., Kobayashi, Y., Ito, S., Nemoto, T., Sakuma, K., Kashima, H., & Mizuno, M. | 2012 | Longitudinal study | Fifty-six people with schizophrenia who were discharged through a deinstitutionalization project were enrolled in this longitudinal study and prospectively assessed with regard to their symptoms, social functioning, and subjective quality of life. The severity of social anxiety symptoms was measured using the Liebowitz Social Anxiety Scale (LSAS). Global/Social functioning and subjective quality of life were evaluated using the Global Assessment of Functioning Scale, the Social Functioning Scale, and the World Health Organization–Quality of Life 26 (WHO-QOL26). | The present findings revealed that social anxiety symptoms were commonly reported among elderly patients with remitted schizophrenia after hospital discharge and that the development of social anxiety symptoms was not associated with psychotic symptoms or social functioning, but with subjective quality of life.  Changes in daily life (e.g. discharge from hospital) could have some negative impacts on subjective quality of life, inducing social anxiety over the long term. The current study demonstrated that subjective quality of life in patients with social anxiety symptoms remained relatively low after a 5-year period.  Another explanation is that a hospitalization period of more than 20 years might cause a reduction in social functioning or social communication skills. Given that the progression of illness also causes cognitive impairment, the elderly patients could not sufficiently cope with their distress during daily life.  Although the degree to which depression is associated with social anxiety remains debatable, aging individuals often experience depression when they encounter social difficulties. Further investigations of the association between social anxiety and depression in aging schizophrenic patients are needed. |
| Este, D.V., & Maglione, J.E. | 2013 | Research article | The unique features of aging that should be taken into account when considering treatment guidelines for the elderly with schizophrenia are explored. | Psychosocial interventions are effective in improving the functioning of older adults with schizophrenia.  Determining the biological pathways underlying cognitive impairment in schizophrenia and developing treatments to improve these deficits in the elderly should be priority areas. |
| Jovelet, G. | 2018 | Observational study | The author retraces the historical journey of psychotic elderly patients - from general hospitals to the hospital then a geriatric hospital. | Psychiatry plays an important role in elderly psychotic patient care. |
| Jovelet, G. | 2010 | Opinion article | On demographic and epidemiologic data base, a situation of patients over 60 years with psychic disorders is carried out. | Clinical positions and medical-economic pressure have a concrete impact on the care of aging psychotic subjects. The retirement home cannot be the only answer to this issue and a range of recent and innovative structures must be proposed. |
| Jovelet, G. & Charazac, P. | 2019 | Opinion article | The condition of people with psychiatric disorders is analyzed in terms of the existential and clinical characteristics of psychosis, social identity, and access to assistance and care after the age of sixty. | The authors argue for the integration of these patients into a psychiatric framework law designed for the decompartmentalization of their social support services and the training of the staff of the nursing homes they are sent to. |
| Kasckow,J.W., and al. | 2001 | Simple comparative study | Comparison of the life quality for patients more than 45 years with schizophrenia depending on whether they are hospitalized (n=54) or outpatients (n=54). Measures realized from Quality of Well-Being Scale of Anderson (1989). | "The PANSS Positive Symptoms, Negative Symptoms, and General Psychopathology subscale scores were all signiﬁcantly higher in the inpatients than in the outpatients. The MMSE scores and QWB scores of the inpatients were lower than those of the outpatients. The groups did not differ, however, on HAM-D scores" (p.71)  "In the inpatient group, a greater severity of symptoms ŽPANSS Positive Symptoms subscale and General. Psychopathology subscale was associated with worse health-related quality of well-being" (p.72) |
| Kermis, M. D. | 1987 | Opinion article | In support of statistic data outcome of research work, conditions of American aging subjects with psychiatric pathology are traced.  "I address issues of equity in the distribution of psychiatric care, policy dimensions affecting this distribution, and projections of the impact of the DRG and RUG systems" (p.270) | Elderly people, in general, are treated in more restrictive and less appropriate contexts than younger persons with mental disorders.  The advent of DRGs and RUGs, the new prospective methods of health care reimbursement, are likely to compound this potentially lethal situation. |
| Laganà, L., & Shanks, S. | 2002 | Literature review | “This article reviews studies conducted within the past three decades on the biases held by mental health professionals (primarily psychiatrists and psychologists) toward older patients and vice versa” (p.271). | It is time to find successful and interdisciplinary ways to improve how older adults and mental health professionals deal with one another. These efforts should creatively enhance the quality of the mental health services offered to older patients, in addition to dispelling age-related myths and corresponding obstacles to the utilization of these needed services by older adults. |
| Lang, P.O., and al., | 2012 | Prospective study | In a medico-psychiatric unit, 150 aging patients hospitalized with mental comorbidities are taken care of "to evaluate the effect of interdisciplinary geriatric and psychiatric care on the appropriateness of prescribing" (p. 406.e1) | “Compared with admission, the intervention reduced the total number of medications prescribed at discharge from 1347 to 790 (P < .0001) and incidence rates for potentially inappropriate medications and PO reduced from 77% to 19% (P < .0001) and from 65% to 11% (P < .0001), respectively. Independent predictive factors for PIP at discharge were being a faller (odds ratio [OR] 1.85; 95% confidence interval [CI] 1.43e2.09) and for PO, the increased number of medications (OR 1.54; 95% CI 1.13e1.89) and a Charlson comorbidity index greater than 2 (OR 1.85; 95% CI 1.38 e 2.13)". |
| Larkin, J. A., and al. | 1992 | Observational study | "This paper […] identifies the needs of elderly people attending Crossroads Community Rehabilitation Program and address how those needs were met" (p.4). | Creating a rehabilitation program adapted to elderly subjects requires the identification of their real difficulties and needs, favoring their empowerment, basing the program on research findings and collaboration with the services involved. |
| Larivière, N., Gélinas, I., Mazer, B., Tallant, B., & Paquette, I. | 2002 | Litterature review / cohort study | Participants were selected from archival data and screened for eligibility by the main investigator. Measures were performed using GAF, Social Functioning Scale, Social Behavior Schedule (SBS), ADL functioning scale, AMPS, MMSE, SLDS, Housing Situation Scale. | The findings of this study demonstrated that participants’ psychiatric, social, and functional status remained stable following discharge from the psychiatric hospital.  In addition, the absence of significant deterioration in overall functioning when transferred to community homes and the satisfaction expressed by the participants regarding their community residence was comparable to other studies with younger samples.  Relocating older adults with severe and chronic mental illness and a long institutional history to the community is a process that requires careful planning and preparation: participants were informed months in advance of their candidacy for a community placement, counseling and visits were organized, family members were invited to be active participants in the preparation process. |
| Leff, J., & Trieman, N. | 2000 | Comparative study | “The total long-stay population of Friern Hospital and several hundred long-stay patients in Claybury Hospital were assessed with a batch of eight schedules while in hospital. They were followed up after one year in the community and then at five years” (p.217). | Of the 670 discharged patients, 126 died before the five-year follow-up. Data were obtained on 523 (97%) of the survivors. There was no change in the patients' clinical state or in their problems of social behavior. However, they gained domestic and community living skills. They also acquired friends and confidants. They were living in much freer conditions and the great majority wanted to remain in their current homes. |
| Limosin, F. | 2009 | Epidemiological study | A national multicentric epidemiological study incorporating a cross-sectional assessment then a prospective cohort follow-up after five years was conducted to estimate causes of mortality in schizophrenic patients at least 60 years of age treated by sectors of adult Psychiatry. | The rate of premature mortality before the age of 65 remains two times higher than the general population. Suicide is the first cause of this mortality but all the natural causes are over-represented as well. |
| Mac Gilp, D. | 1991 | Observational study | An investigation of 48 hospitalized patients between 1 and 41 years who were recently let out of hospital was carried out to identify their feelings/perception about their deinstitutionalization. "The questionnaire in this study covered the quality of life areas — living situation, family and social relationships, leisure, work, finance, health and safety — as well as satisfaction with the CPN" (p.1207) | Each respondent (48 100%) preferred to live in the community rather than being in a mental hospital Most respondents (46, 95%) were satisfied with life in general. Twenty-eight were visited by community psychiatric nurses and 24 (85%) were satisfied with the care provided. |
| Martens, P.J, and al. | 2007 | Retrospective cohort study | “Using unidentifiable administrative records (1997-1998 to 2001-2002) from the Population Health Research Repository housed at the Manitoba Center for Health Policy, the authors determined the 5-year prevalence of people aged 55 and over for 3 categories of mental illness: cumulative mental disorders, all mental illnesses and dementia” (p.581) | “From the group aged 55 to 59 years to the group aged 90 years or older, the prevalence of mental illness increased with the population’s age. The prevalence of any mental illness rose from 32.4% to 45.0% in men and from 42.6% to 51.9% in women, and dementia prevalence rose from 2.0% to 33.6% in men and from 1.3% to 40.3% in women.” (p.581) |
| Martin, N., & Johnston, V. | 2007 | Retrospective cohort study | “Using nonidentifying administrative records (fiscal years 1997–1998 to 2001–2002) from the Population Health Research Data Repository housed at the Manitoba Centre for Health Policy, we determined the 5-year period prevalence for individuals aged 55 years and over (119 539 men and 145 752 women) for 3 mental illness categories: cumulative mental disorders, any mental illness, and dementia. We calculated age-specific and age-adjusted rates of home care and PCH use and the prevalence of mental illness in PCH residents” (p.581) | The authors note that mental disease rose according to the age of the population: “The prevalence of any mental illness rose from 32.4% to 45.0% in men and from 42.6% to 51.9% in women, and dementia prevalence rose from 2.0% to 33.6% in men and from 1.3% to 40.3% in women” (p.581). However, membership in one of the 3 categories of mental disease is different for each year observed by the authors. |
| Martinsson, G.,and al. | 2012 | Retrospective cohort study | “Data concerning drug treatment for older persons from 2006 to 2008 was gathered from the Swedish Prescribed Drug Register. Mental disorders, were evaluated in order to identify associated drugs”. 188 024 older individuals were included’’ (p.581) | “Physicians specialized in older persons’ disorders and mental health were rarely the prescribers of these drugs. The results indicate a future need of more specialists in geriatrics and psychiatry” (p.581). |
| McInerney, SJ., and al. | 2009 | Observational study | This study adds to the previous work carried out on patients discharged from large psychiatric hospitals into the community.  87 long-term psychiatric patients were assessed on four standardized assessment instruments designed to assess their attitudes towards community life and the level of functioning in the community. | Patients expressed a desire to continue to live in the community and while they showed improvements in self care and social functioning in the first year following discharge, these improvements were not sustained after 5 years in the community. Further training is needed for staff in the community residences so that patients can achieve their maximum potential. |
| Meehan, T., Robertson, S., Stedman, T., & Byrne, G. | 2004 | Longitudinal study | All patients (n = 60) who were relocated to the ECUs were assessed using a number of standardized clinical and general functioning instruments at 6 months and 6 weeks pre-move, and again at 6 weeks, 6 months and 18 months post-move. | The mortality observed in the follow-up period (21,6%) is likely to be related to physical ill health and old age rather than the trauma associated with relocation. While overall functioning did not improve following relocation, patients had more independence and greater access to community-based activities.  Following relocations an improvement in both social contact and community access and physical health problems gradually increased. |
| Mosher-Ashley, P.M., and al. | 1991 | Observational study | The administrators of 92 nursing homes and rest homes in Western Massachusetts were surveyed on their experiences with elderly residents, with a history of psychiatric hospitalization and their willingness to admit such individuals in the future.  “Each administrator was interviewed via telephone by an interviewer who was not informed of the objectives of the study” (p.243) | Three quarters of the administrators reported that they did not have the support services that the deinstitutionalized elders in their facilities needed. A comparison of the services reported to be important and those reported to be available suggest that simply increasing the availability of psychiatric support services would probably not influence administrators to admit elders with chronic mental illness in the future. |
| Nakamura, R., and al. | 2019 | Longitudinal study | Longitudinal analyzes were performed to compare the effects of the IMR program on clinical symptoms and cortical thickness in the superior temporal gyrus (TSG) in schizophrenic patients whose average age was 61.4 with an average length of hospital stay of 13.1 years at the time of enrollment. | This is the first report demonstrating the effectiveness of the IMR program for improving psychotic symptoms and psychosocial function and protecting brain structure in middle-aged and older inpatients with schizophrenia hospitalized for long periods. |
| Nemoto, T., Niimura, H., Ryu, Y., Sakuma, K., & Mizuno, M. | 2014 | Longitudinal study | Patients were monitored for 5 years and underwent annual examinations that included measures of cognition, psychiatric symptoms, and social functioning. Fifty-six patients completed all the assessments. Measures of cognition included the Letter Cancellation Test as a measure of attention, the Digit Span (DS) of the WAIS-R as a measure of attention/working memory, the Rey–Osterrieth Complex Figure Test as a measure of memory, the Word Fluency Test as a measure of executive function, the Trail Making Test Part A as a measure of processing speed, and the Mini-Mental State Examination as a measure of global cognition. The Positive and Negative Syndrome Scale was used to assess psychiatric symptoms, and the Social Functioning Scale was used to assess social functioning. In addition, the Global Assessment of Functioning was used to measure global functioning. Clinical assessments and cognitive tests were administered at baseline and annually for 5 years | The results suggested that even patients with schizophrenia who have been chronically hospitalized could show a certain degree of improvement in some cognitive deficits after living in the community following their discharge from hospital, although the changes in the variables were relatively small. The present results suggest that even impairments in executive function and verbal fluency may be partly improved by transitioning chronically hospitalized patients to community-based living. It is reasonable to think that appropriate community services, in addition to living in a community, might have also contributed to the improvements in cognition seen in the subjects.  Global cognition and processing speed improved for a few years and then began to decline thereafter, although the patients' symptoms and global functioning improved consistently. This change might represent a genuine decline, and aging might have also influenced this decline.  Psychiatric symptoms and global functioning almost consistently improved over the 5-year follow-up period. |
| Niimura, H., and al. | 2011 | Observational study | Fifty-seven middle-aged and elderly schizophrenic patients with successful aging were identified using the attitude towards aging scale, the scale for preparing for behavior in old age and assessments of their cognitive function, psychiatric symptoms, social functioning and quality of life. Multiple regression analysis was used to detect the determinants of attitude toward aging. | The multiple regression analysis revealed that quality of life was a significant determinant: a higher quality of life was related to a more positive attitude towards aging and less active preparing behavior. The significant predictors of preparing behavior were quality of life and the length of the hospital stay: a longer hospital stay and a higher quality of life were related to less active preparing behavior. |
| Nikkonen, M. | 1997 | Observational study | Description and analysis of the lifestyle of deinstitutionalized long-term psychiatric patients in northern Finland. Data was collected by interviewing 25 released long-term psychiatric patients. | The results suggest that home was the central area of ​​life for outpatients, but they differed in their relationship to it. For some, the house was a refuge to hide from social life. Elderly patients who generally viewed work as a virtue found it difficult to find something to do in their modern suburban homes. Social participation was mainly marked by the old familiar hospital models. |
| Nikkonen, M. | 1995 | Observational study | This article describes and analyzes, from a socio-cultural point of view, the lifestyles of 25 patients aged 42 to 70 years hospitalized between 3 and 30 years in psychiatry and released in the north of Finland. The paper focuses on subjective control of life and fundamental experiences in life. The data consist of interviews with 25 outpatients who have moved to live in residential homes, rehabilitation centers or their own homes after receiving preparatory training at the psychiatric hospital. | The findings suggest that the way of life of the outpatients did not significantly differ from that of other people belonging to the same generation of Finns. With respect to control of life, a conspicuous feature was the strong tendency to let themselves be led by others, and to self-sacrifice for the good of others. For the patients, independence was the best aspect of open care. Fear for being branded as a psychiatric case or as a former mental patient threatened the patients' external control of life. The central element of control of life for Finns is work. The outpatients thought work a matter of honor and they did not find their present, inactive way of life satisfactory. |
| Pancrazi, M., and al. | 2015 | Opinion article | Inventory of obstacles which slow down the development of the provision of care for elderly people with psychiatric pathology. | The issues for the future consist of further refining the collection of data on the needs of the ageing population to better dimension the responses in matter of care and organization.  Territorially, coordination of actions with existing arrangements in particular, the geriatric sector and the networks must be analyzed to avoid overlap. |
| Perrin-Haynes, J. | 2011 | Observational study | Based on a national survey, this study presents the main information for 2007 on diseases which elderly people living in institutions like geriatric hospital, nursing homes, etc. suffer from. | Eight residents out of ten present at least one neuro-psychiatric infection.  Residents combine a total of 6 pathologies including psychiatric pathologies and consume 6.5 tablets in a day. |
| Rahman, M., and al. | 2013 | Observational study | “To estimate the effect of a nursing home’s share of residents with a serious mental illness (SMI) on the quality of care. Data Sources. Secondary nursing home level data over the period 2000 through 2008 obtained from the Minimum Data Set, OSCAR, and Medicare claims. Study Design. We employ instrumental variables approach to address the potential endogeneity of the share of SMI residents in nursing homes in a model including nursing home and year fixed effects” (p.1279) | “An increase in the share of SMI nursing home residents positively affected the hospitalization rate among non-SMI residents and negatively affected staffing skill mix and level. We did not observe a statistically significant affection inspection-based health deficiencies or the hospitalization rate for SMI residents” (p.1279) |
| Ramaprasad, D., and al | 2015 | Retrospective cohort study | Study measuring the level of disability and the quality of life of the elderly suffering from chronic and persistent mental illnesses (PMI) compared to an elderly population without illness (CG). 200 elderly people with mental illness attending psychiatric services were studied. A comparison group of 103 healthy elderly people was drawn from the same study area as the control group. They were assessed using WHO-DAS and WHOQOL-BREF. | People with chronic mental illness continue to suffer from psychiatric disabilities at an advanced age, which cannot be attributed to normal aging. The level of disability has a negative impact on their quality of life. |
| Raucher-Chéné, D., and al. | 2011 | Literature review | Summary of current clinical and cognitive knowledge concerning schizophrenic subjects over 65 with the aim of better understanding the changes inherent in the elderly. Proposal for a comprehensive study of the management of this population in order to raise awareness among the general population in the coming years. | Develop care nets between psychiatrists, geriatricians and actors in the medico-social field.  Geriatric psychiatry must be able to fulfill this role of care coordination.  Encouraging research to continue its work on ageing subjects with schizophrenia. |
| Reinharz, D., and al. | 2000 | Retrospective cohort study | A retrospective cohort of 96 pairs of psychiatric hospital patients with 1 member deinstitutionalized between 1989 and 1998 was monitored for nearly 10 years. All use of medical and social services was documented. Patients were evaluated at the start and end of the study on various clinical and social dimensions, as well as on quality of life. | Deinstitutionalized patients obtained more positive results than those who were still hospitalized. The lower cost of living for deinstitutionalized patients easily compensates for the cost of services required for treatment in the community. Deinstitutionalization, as implemented in the hospital studied, remains a cheaper option for many patients than continuing hospitalization and is likely to improve their mental state and well-being. |
| Rollet, A. | 2014 | Opinion article | Questioning about the place to be given to the psychiatric age of the elderly. Proposal for thought in support of the paradoxes of the criteria of care and age which are often opposed and insufficiently combined in terms of the institutional issues they raise. | The middle way needs to be found in our institutional clinical practice between invisibility and discrimination which are both forms of exclusion. Between complete ignorance and segregative instrumentalization, age needs to be given a reasonable and human place and conceive of the idea that the ageing person is not a special case of the adult. |
| Ryu, Y., Mizuno, M., Sakuma, K., Munakata, S., Takebayashi, T., Murakami, M., Falloon, I. R. H., & Kashima, H. | 2006 | Longitudinal study | Seventy-eight patients with schizophrenia transferred to a community facility. All evaluations were performed prior to the patients’ discharge and were repeated 12 and 24 months after discharge using the Positive and Negative Syndrome Scales, the Global Assessment for Functioning, the Schedule for Assessment of Insight, the Rehabilitation Evaluation Hall and Baker Scale, the Social Functioning Scale, the Drug Attitude Inventory, and the Mini-Mental State Examination. | Two years of community living resulted in a significant reduction in psychiatric symptoms, not only in positive symptoms but also in negative and general symptoms.  A significant improvement in global functioning was also observed during the 2-year study period (before discharge, 58.6; 2 years after discharge, 67.0; p < 0.001).  Unexpectedly insight, as assessed using SAI, gradually deteriorated.  The Social Activity, Speech Skills, Disturbed Speech, Self-Care and General Behavior scores improved significantly between the discharge and 24-month scores.  After 24 months of living in the community, Withdrawal, Independence (Performance), Independence (Competence) and Employment improved significantly.  The overall neuroleptic dosage remained unchanged over the 2 years as did the drug attitude of patients.  The MMSE score remained unchanged over the 2-year follow-up period. |
| Salime, S, and al. | 2019 | Observational study | Between June 2017 and December 2018, authors used the methodology of free and hierarchical associations of Abric and Verges to observe 790 health professionals in the Grand Est Region of France. | Analysis of the data gathered revealed that the following elements were characteristic of the central core of this social representation - "chronic", "dependent", "behavioral disorders", "memory disorders", "loneliness" and "social isolation".  These results thus highlight that the SR of health professionals is substantially identical to that of the global SR on several dimensions. |
| Salisbury, t., and al. | 2017 | Comparative study | The quality of care was assessed in 193 longer-term hospital and community facilities in Bulgaria, Germany, Greece, Italy, the Netherlands, Poland, Portugal, Spain and the United Kingdom. Data on user care assessment were collected from 1,579 users of these services. The country-level variables were compiled from publicly available data. Multilevel models were adapted to assess associations with quality of care and the care experiences of service users. | Significant positive associations were found between deinstitutionalization and (1) five of seven quality of care domains, and (2) service user autonomy. A 10% increase in expenditure was associated with projected clinically important improvements in quality of care.  Greater deinstitutionalization of mental health mental health services is associated with higher quality of care and better service user autonomy. |
| Schmidt, L.J., and al. | 1977 | Retrospective cohort study | Using data collected regularly to review the use of Medicaid, the authors studied the characteristics of patients in Utah psychiatric nursing homes.  “After matching the records for each patient, two research files were created: (1) a one-point file that contained one record for each of 3,803 patients, with a review date between January 1973 and January 1974, and provided cross-sectional, one-point-in-time comparisons; and (2) a two-point file that contained two records per patient, one prior to and one after March 1973, and was used to investigate changes in patient status and behavior.” (p.688) | Most psychotic patients were significantly younger than their non-psychiatric counterparts. Over time, all patient groups showed an increase in prescribed psychoactive drugs and a decrease in activity. The consequences of this model of care raise serious questions about our current dependence on nursing homes for the care of the psychiatric patient. |
| Shadish, W.R., & Bootzin R.R. | 1981 | Opinion article | The authors examined 3 concepts which are at the heart of deinstitutionalization: “First, as reflected in the numbers of patients placed and the dollars spent, nursing homes are the centerpiece of a de facto mental health policy of institutionalization (Kiesler, in press). Second, the challenge to concepts of the community-based care movement can be made parsimoniously, yet forcefully, using nursing homes as an example. Of course, the arguments generated here can often be applied with equal force to other settings. Finally, nursing homes can be used to initiate some positive changes in mental health policy that can affect large numbers of mental patients” (p.488) | Nursing homes offer a unique opportunity to improve the system of large-scale mental health care. This opportunity will be missed if we continue to allow administrative influences of well-being to shape health policy for a large number of chronic patients. Retirement homes can be a solution if these implement skills developed in mental health care. |
| Silva, P. R. F. da, Carvalho, M. C. de A., Cavalcanti, M. T., Echebarrena, R. C., Mello, A. S. de, Dahl, C. M., Lima, D. B. de, & Souza, F. M. | 2017 | Longitudinal study | The research model used was longitudinal follow-up of the deinstitutionalized population with baseline assessments (immediately before leaving the hospital) and two more assessments in month 9 and in month 18 after the baseline assessment. This article discusses the baseline and nine-month assessments. To analyze the effects of the CTI intervention, the population was randomized by cluster (household). The population was split into two groups. In the first, Critical Time Intervention was added to the institution’s program. The other group (control) received only the institution’s standard of care. | Analyzing the assessments of the 71 patients showed significant evolution in social and personal performance.  Results showed a negative influence of age on the evolution of the overall PSP score, indicating that younger patients (under the age of 70) could benefit more from the deinstitutionalization program offered. |
| Sommers, I., and al., | 1988 | Comparative study | The factors associated with the placement of 248 elderly (≥ 60 years) deinstitutionalized mentally ill patients were examined in four contexts: independent community programs, level III, and nursing homes level IV. The objective was to examine the process of deinstitutionalization of this population. | The risk of institutionalization increased as cognitive skills and family support decreased. The most consistent predictor of placement was past residence. Surprisingly, the number of medical conditions and the need for nursing or therapeutic services were not important predictors of nursing home placement. |
| Talbot, J.A. | 1983 | Opinion article | This article details what we now know about the care and treatment of the chronically mentally ill. It discusses the number of chronically mentally ill people, their needs, effective treatment, barriers to successful care, system problems and finally economic problems. | Deinstitutionalization was initially driven by noble intentions - treating and caring for the mentally ill in environments closer to their homes, families and neighborhoods; treating people in more therapeutic and less restrictive contexts; and provido,h the range of services and settings in the community rather than in remote institutions. However, few of these intentions have been realized. |
| Thiebaux, J.F, & Kardache, F. | 2014 | Opinion article | Resumption of an original experiment for elderly people in the MAS CAREIRON hospital center in Uzès, France with its his geronto- psychiatry unit and the ESSPER device (Stable Community Care Team Burst and Reactive). | Access to psychiatric care for elderly with mental pathology must be responsive to a care organization entering a well-coded geronto-psychiatric sector. |
| U.S. Department of Health and Human Services. | 1999 | Literature review | "The Office of the Surgeon General" analyzed 3000 articles and testimonies of experienced people in the field of mental health, elderly people and mental disorders. | The main results are that literature agrees on effectiveness of mental health management and that there are treatment options for almost all mental disorders. |
| Uyeda, M.K., and al | 1987 | Comparative study | This article focuses on federal policies that affect the financing of mental health care services. The programs discussed are Medicare, the federal insurance program for the elderly and disabled, and the Civilian Health and Medical Program of the Uniformed Services (CHAMPUS), the Department of Defense program for military dependents and retirees. | Efforts to control the costs of care often result in the policy decision to significantly limit the scope of practice of non-physician health care providers when compared to that authorized by state-level practice and licensing laws. |
| Vahia, I., & Bankole, A. | 2007 | Literature review | This review provides a comprehensive overview of schizophrenia in the elderly. The authors examined the epidemiology, classification and psychopathology of schizophrenia and schizophrenia later in life to ensure clinical care, public policy, and future research. | About half of the elderly with schizophrenia have favorable results in each symptom and social category. The number of clinical research programs with large databases must be enlarged in order to access the generalization of results. |
| Voyer, and al | 2003 | Opinion article | Evidence is examined for the use of psychotropic medications as a viable treatment option for the elderly experiencing mental health challenges both in the community and in the long-term care setting. Alternative non-pharmacological approaches that nurses can use to augment care are also briefly discussed. | High-quality mental health nursing care of elderly clients is a goal that does not seem to have yet been widely achieved. Nurses have the skills to play a significant role in the initial assessment and treatment of mental health problems experienced by the elderly. It is possible that unfortunate outcomes such as hospitalization which are associated with the misuse of psychotropic drugs could be decreased by high-quality nursing care. |
| World Health Organization. | 2008 | Observational study | “The WHO Regional Office for Europe" developed a questionnaire of 90 questions on mental health that 42 participating countries completed. | “The large majority of countries now have mental health policies and legislation, and many, but not all countries are making some progress towards implementing community-based mental health services”, “most countries are creating an increasingly diverse and competent workforce”. (p. 179) |
| World Health Organization. | 2013 | Global action plan | 4 objectives are defined: "strengthen leadership and governance in the field of mental health; provide comprehensive mental health and social support services, integrated and adapted to needs in a community setting; implement promotion and prevention strategies in the field of mental health; strengthen information systems, evidence base and research in mental health" (p.10) | The authors make proposals for implementing the action plan and also provide indicators of change for each recommendation. |
